# Impact of the season and prevalence of intestinal parasitosis at the Notre Dame de l’Espérance University Hospital Center, Democratic Republic of the Congo

**DOI:** 10.12688/f1000research.160135.3

**Published:** 2025-10-06

**Authors:** lufuluabu mpemba Alphonse, Tshishimbi kalala Jean Hubert, Tshodi bulanda Arsène

**Affiliations:** 1 internal medecine, University of Mbuji-Mayi, Mbuji-mayi, Kasai oriental, Democratic Republic of the Congo; 2 pediatrics, University of Mbuji-Mayi, Mbuji-Mayi, Kasai oriental, Democratic Republic of the Congo; 3 gynecology obstetric, university of Mbuji-Mayi, Mbuji-Mayi, Kasai oriental, Democratic Republic of the Congo

**Keywords:** University of Mbujimayi/Intestinal Parasitosis/Season/ Association/Protozoa/Helminths

## Abstract

**Background:**

Intestinal parasitosis are one of the main causes of morbidity and mortality in Africa. The tropical climate in the D.R. Congo provides parasites with an environment conducive to their proliferation. The prevalence rates of intestinal parasitosis remain poorly understood in the D.R. Congo.

**Objective:**

This study aims to estimate the overall and specific prevalence of intestinal parasitosis and to determine an association between intestinal parasitosis and the season at CHUNDE.

**Methods:**

From January 1, 2020, to December 31, 2021, patients for whom a direct stool examination was requested at the Notre Dame de l’Espérance University Hospital Center were included in this study. Stool samples were collected and examined under an optical microscope.

**Results:**

During the period of the study, we recorded 187 patients aged 2 to 77 years. The prevalence of intestinal parasitosis was 75.40%. The specific prevalence rates for parasites were as follows: *E. histolytica/dispar*, the most common, with a prevalence of 55.08%, followed by *P. hominis* and *G. lamblia* with respective prevalence rates of 9.09% and 6.24%. *A. lumbricoide* had a prevalence of 27.81%, followed by *S. mansoni*, *A. duodenalis*, and *E. vermicularis* with respective prevalence rates of 3.74%, 1.60%, and 1.07%. There was no association between the season and the overall prevalence of intestinal parasitosis.

**Conclusion:**

The prevalence of intestinal parasitosis was higher amang symptomatic patients at CHUNDE. There is no statistically valid association between the season and the prevalence of intestinal parasitosis.

## Introduction

Intestinal parasitic infections are serious diseases worldwide. However, doctors working in these regions tend to give them little attention due to the commonality of their occurrence. As a result, they are rarely a reason for regular consultation and are often neglected. ^1, 2^ Although they attract little interest today compared to diseases such as AIDS, tuberculosis, malaria, and onchocerciasis, they remain a public health issue in tropical and impoverished areas. ^3^

Climatic conditions are one of the main factors that contribute greatly to the spread of intestinal parasitic infections, by increasing their transmission and perpetuating the parasitic cycles. ^4^

According to World Health Organization (WHO) estimates, more than three billion people are affected, with 450 million severely ill; of these, over 50% are school-aged children. ^5^ The global prevalence is 35.8% in the world population. ^6^ These diseases have disastrous health, social, and economic consequences for more than one billion people. ^6^

In Africa, a study conducted in Morocco in 2019 among migrants from sub-Saharan Africa (Equatorial Guinea “30.6%”, Côte d’Ivoire “16.6%”, Senegal “9.29%”, Cameroon “8.01%”, with the remaining patients coming from Niger, Togo, Congo, Burkina Faso, Mali, Gabon, and Benin “35.5%”) showed a prevalence of 43.18% for intestinal parasitic infections. Among these individuals, 63.15% were infested with various digestive parasites simultaneously. ^7^ A study conducted Algeria showed a highly significant difference of prevalence between symptomatic (82.3%) and asymptomatic subjects (14.9%), with the majority attributable to protozoan infection. The most common species in the symptomatic subjects were *Blastocystis* spp. (43.8%), *Entamoeba histolytica/dispar* (25.4%) and *Giardia intestinalis* (14.6%) and more rarely *Enterobius vermicularis* (02.1%), *Teania* spp. (0.6%) and *Trichuris trichiura* (0.2%), while in asymptomatic population *Blastocystis* spp. (8%), *Entamoeba coli* (3.3%) and *E. histolytica/dispar* (2.5%) were the most common parasites detected with no case of helminth infection. ^8^ Another study conducted among schoolchildren in the Republic of Congo revealed a prevalence of intestinal parasitosis of 25.7%, with the most frequent parasites being: *Entamoeba coli* (52.2%), *Ascaris lumbricoides* (13%), and *Endolimax nana* (10.9%). ^9^

In the DRC, data on intestinal parasitosis are poor. The few available data that parasitic infections pose a public health problem due to multiple crises that have led to displacement and a lack of potable water, food hygiene, and sanitary facilities. ^10^ A study conducted in Bukavu in 2016 on the impact of seasons on intestinal parasitic infections showed a prevalence of 94%. The identified helminths, in decreasing order of frequency, were: *S. mansoni* (30.6%), *S. stercoralis* (21.3%), *Ancylostoma duodenales* (13.6%), *A. lumbricoides* (12.6%), *T. trichiura* (9.0%), and *T. saginata* (6.6%). Identified protozoa included *Pentatrichomonas hominis* (13.69%), *Entamoeba histolytica* (6.75%), and *Giardia lamblia* (4.76%). ^11^ According to a study conducted in Kinshasa more than half of the diagnosed individuals were infested with *A. lumbricoides* at 58.3%, followed by 30% of individuals hosting *E. histolytica*, and *A. duodenales* was isolated in 11.7% of the diagnosed individuals. ^12^

The East Kasai region is dominated by a humid tropical climate with two seasons (dry season from 15 ^th^ May to 15 ^th^ August and rainy season from 15 ^th^ August to 15 ^th^ May). ^13^ The population living in the eastern part of its capital, Mbujimayi, and the surrounding villages, served by CHUNDE, is poor and lives in very poor hygienic conditions. We have not identified any study that could inform us about the prevalence of intestinal parasitic infections here. The prevalences of intestinal parasitosis as well as the factors that influence them are poorly documented in East Kasai.

The prevalence of intestinal parasitic infections is likely very high at CHUNDE and may be associated with the season.

### General objective

To determine the prevalence of intestinal parasitic infections via stool examination at CHUNDE and to assess the impact of the season on the frequency of intestinal parasitic infections in general and on each specific type.

### Specific objectives

• To determine the specific prevalence of each identified intestinal parasitic infection at CHUNDE.
• To determine the prevalence of intestinal helminths and protozoa at CHUNDE.
• To determine the association between the season and the prevalence of intestinal parasitic infections.

## Methods

### Study population

This study included patients who consulted at the Notre Dame de l’Espérance University Hospital Center (CHUNDE) and were requested to have a direct stool examination. This center serves the East Kasai province, particularly the rural-urban areas to the east of Mbujimayi city in the DRC. These areas are characterized by poor sanitary and economic conditions. The study period was from January 1, 2020, to December 31, 2021.

### Sampling and type of study

Thid is a cross-sectional study with recruitment of patients consecutively meeting the inclusion criteria.

### Inclusion and exclusion criteria

The study included all patients who consulted at CHUNDE during the study period and were requested by physicians to have a direct stool examination. Patients who did not provide a sample for this examination were excluded.

### Sample collection

Fresh stool samples of approximately 10-20 grams (5-6 milliliters if liquid) were collected by the patient. For this collection, the laboratory provided each patient with a clean container and rod. The patients were provided with these instructions: avoid touching the inside of the container; if the stools are associated with mucus or blood, preferably collect them; prevent urine and stool from mixing.

### Direct stool examination

The direct stool examination was conducted within 30 minutes of sample collection. Approximately 1 gram of stool were mixed with 1drop of saline solution (0.9%) on a slide. After homogenization, this preparation was covered with a slide and observed under an optical microscope at 10x and 40x objectives. The results were recorded in the laboratory register and entered into an Excel database as follows: Parasitic infection (yes, no), type of parasite (helminths, protozoa), multiple infestation (yes, no), types of combined parasites, *E. histolytica* (yes, no), *G. lamblia* (yes, no), *P. hominis* (yes, no), *A. lumbricoides* (yes, no), *S. mansoni* (yes, no), *A. duodenales* (yes, no), *E. vermicularis* (yes, no). Cases of *E. histolytica* also included those of *Entamoeba dispar* due to the difficulty in distinguishing them microscopically. Other patients information were obtained directly from the laboratory voucher and recorded in the laboratory register.

### Statistical analysis

The prevalence of intestinal parasitic infections was calculated by dividing the number of patients with at least one identified parasite in a direct stool examination by the total number of patients who underwent this examination. The prevalence in each population group was calculated by dividing the number of patients in that group with at least one identified parasite by the total number of patients in that group who underwent this examination. The prevalence of a specific intestinal parasitic infection was calculated by dividing the number of patients with a specific parasite by the total number of patients who underwent the examination. The confidence interval (CI) for each prevalence rate is 95%. The relationship between the prevalence of parasitosis and climate was studied using the Yatt corrected chi-square test and Fisher as appropriate. The test was considered valid if the P value was less than 0.05. Statistical analyses were performed using EPI INFO 7.2.6.0 and R version 4.3.3.

### Ethical considerations and informed consent

This study received approval from the ethics committee of the University of Mbujimayi on N52/CEUM1209 of 11th December 2019. It was conducted in accordance with the requirements of good clinical practices and the principles of the Helsinki Declaration of the World Medical Association, along with any subsequent relevant amendments.

All patients who have participated in this study have provided their consent by signing a written Document, the consent form, or their guardian, where applicable.

## Study results

### Characteristics of the study population

From January 1, 2020, to December 31, 2021, 187 patients were included in our study.

Table 1 summarizes the demographic characteristics: Among the 187 patients included in our study, 94 were female and 93 were male. The sex ratio was one woman for one man. A total of 158 patients were adults [≥18 years <70], 20 were children [<18 years], and 9 were elderly [≥70 years]. The mean age was 41.68 ± 17.95 years. The youngest patient was 2 years old, and the oldest was 77 years.

**Table 1.** Demographic characteristics of patients undergoing direct stool examination between 2020 and 2021.

	Frequency (N=187)	%
**Sex**		
Female	93	49.73
Male	94	50.27
**Age groups**		
Children (<18 years old)	20	10.69
Adults (18-70 years old)	158	84.49
Senior adults (˃70 years old)	9	4.81
Mean age	41.68±17.95	

**Figure 1.**
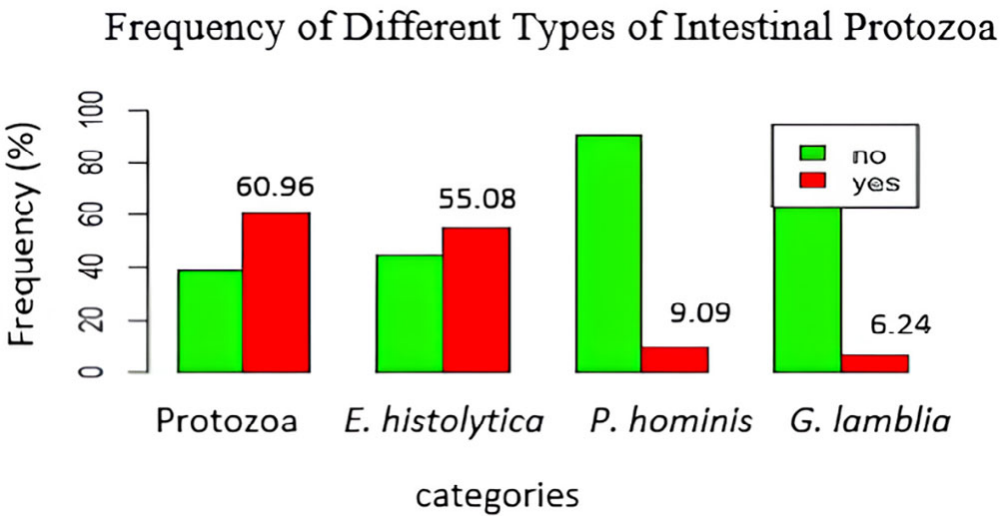
Frequency of different types of intestinal protozoa.

### Frequency of intestinal parasitic infections

As shown in Table 2, a total of 141 patients, or 75.40%, had at least one intestinal parasitic infection. Forty-seven patients (24.13%) had multiple infestations, of which 37 (19.79%) combined at least one helminth and one protozoan, and 10 (5.35%) combined at least two protozoa. The most frequent combination was *E. histolytica* and *A. lumbricoides*, with 27 cases (14.44%). Intestinal parasitic infections were more frequent among children (95.00%) and elderly adults (88.89%). They were also more common in women (78.49%) than in men (72.34%). A total of 114 patients (60.96%) had at least one intestinal protozoan, and 66 patients (35.29%) had an intestinal helminth infection.

**Table 2.** Frequency of intestinal parasitic infections, intestinal helminth infections, and intestinal protozoa.

	Frequency (N=187)	%
**Intestinal parasitic infections**	141	75.40
**Type of parasitosis**		
Protozoa	114	60.96
Helminths	66	35.29
**Multiple infestation**		
Yes	47	24.13
No	94	50.27
**Different combinations**		
*Protozoa-helminth *	37	19.79
*E. histolytica/dispar A. lumbricoides*	27	14.44
*E. histolytica/dispar A. duodenales*	1	0.53
*E. histolytica/dispar S. mansoni*	2	1.07
*E. histolytica/dispar A. lumbricoides S. mansoni*	1	0.53
*E. histolytica/dispar P. homimis A. lumbricoides*	1	0.53
*G. lamblia P. hominis A. lumbricoides*	1	0.53
*G. lamblia P. hominis S. mansoni*	1	0.53
*P. hominis A. lumbricoides*	2	1.07
*P. hominis S. mansoni*	1	0.53
*Protozoa2*	10	5.35
*E. histolytica/dispar P. hominis*	2	1.07
*E. histolytica/dispar P. hominis G. lamblia*	4	2.14
*G. lamblia P. hominis*	4	2.14
**Age group**		
Children	19 (n=20)	95.00
Adults	114 (n=158)	72.15
Senior adults	8 (n=9)	88.89
**Sex**		
Male	68 (n=94)	72.34
Female	73 (n=93)	78.49

### Frequencies of intestinal protozoa

Figure 1 shows that *E. histolytica/dispar* was the most frequent protozoan, with 103 cases (55.08%), followed by *P. hominis*, and *G. lamblia* with respective prevalence rates of 9.09%, and 6.24%.

### Frequencies of intestinal helminths

**Figure 2.**
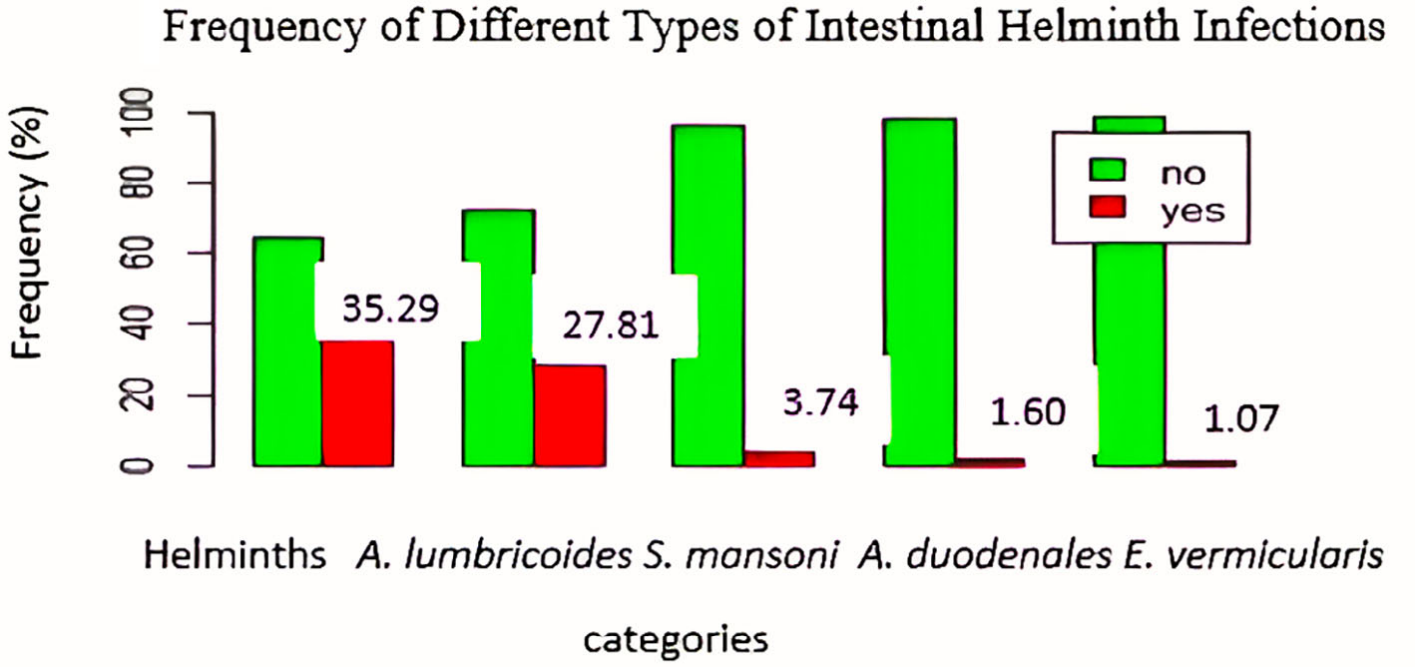
Frequency of different types of intestinal helminth infections.

As shown in Figure 2, *A. lumbricoides/dispar* was the most frequent helminth, with 52 cases (27.81%), followed by *S. mansoni*, with 7 cases (3.74%), *A. duodenale*, with 3 cases (1.60%), and *E. vermicularis*, with 2 cases (1.07%).

### Frequency curves of intestinal parasitic infections

**Figure 3.**
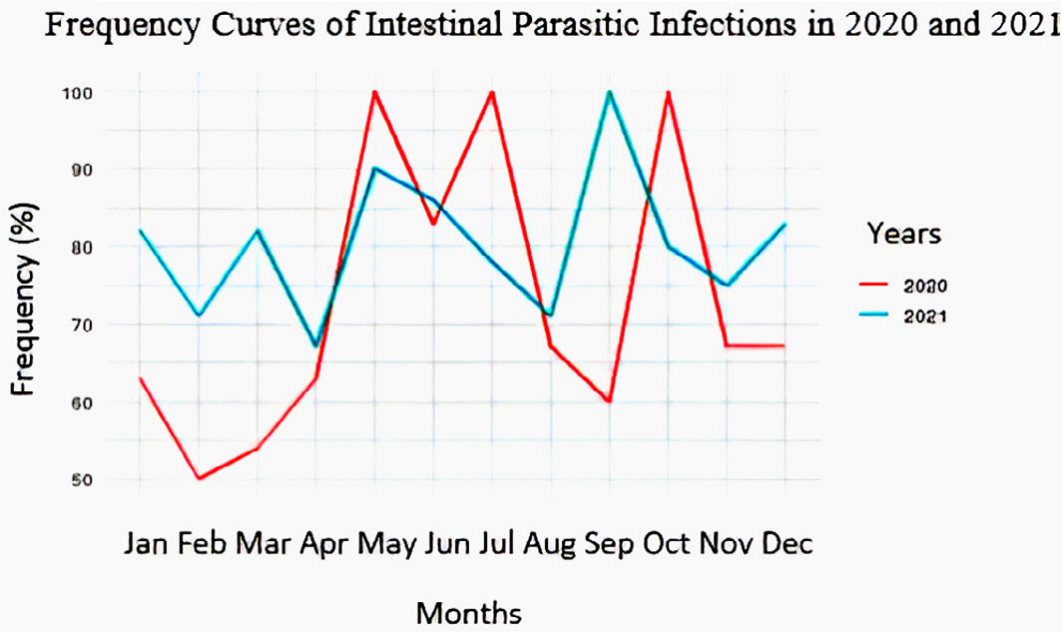
Frequency curves of intestinal parasitic infections in 2020 and 2021.

Figure 3 shows the evolution of frequency of intestinal parasitosis by year (2020 and 2021). It allows to analyze this evolution in relation to the season (dry season: from 15/5 to 15/8 and rainy season: from 1/1 to 14/5 and from 16/8 to 31/12).

In 2020, the frequency of intestinal parasitic infections was high, with peak values during the dry season (83-100% in May, June, and July) and in October. In 2021, the frequency also remained high, particularly with peak values observed during the dry season (87-90% in May and June) and in September.

### Intestinal parasitosis prevalence and seasonal relationship

Table 3 shows that the frequency of intestinal parasitic infections was higher during the dry season (83.33%) compared to the rainy season (72.66%). When considered by year, the frequency was higher during the dry season (87.50%) than during the rainy season (62.69%) in 2020, whereas in 2021, the rainy season recorded a slightly higher frequency than the dry season, with 81.94% and 79.17%, respectively. But the chi-square test showed that there was no association between intestinal parasitic infections and the season.

**Table 3.** Distribution of intestinal parasitic infection cases by year and season.

	Frequency	%
**Rainy season (2020-1)**	(n=139) 101	72.66
2020	(n=67) 42	62.69
2021	(n=72) 59	81.94
**Dry season (2020-1)**	(n=48) 40	83.33
2020	(n=24) 21	87.50
2021	(n=24) 19	79.17
Chi-square	1.6531	
P Value	0.1985	

### Frequency curves of intestinal protozoan infections

**Figure 4.**
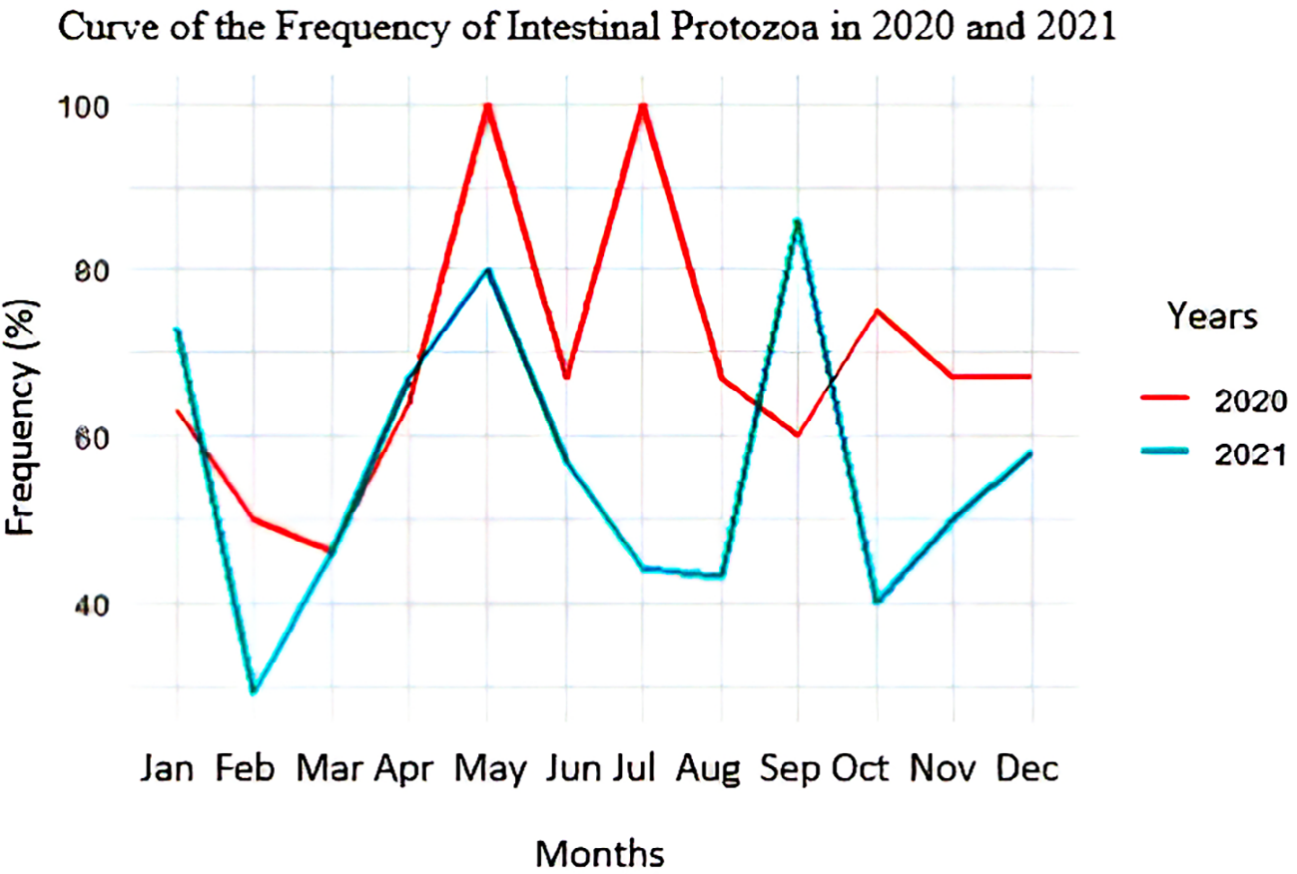
Curve of the frequency of intestinal protozoa in 2020 and 2021.

Figure 4 shows that the frequency of intestinal protozoa was high in both 2020 and 2021. In 2020, two peaks of this frequency were observed during the dry season (100% in May and July). In 2021, two high values of this frequency, 80% and 85%, were observed in May and September, respectively.

### Intestinal protozoan prevalence and season relationship

As shown in Table 4 the dry season recorded a higher frequency of intestinal protozoa (64.58%) compared to the rainy season (59.71%). This superiority remained stable in both 2020 and 2021. There is no association between the frequency of intestinal protozoa and the season. The frequencies of *E. histolytica/diapar*, *P. hominis*, and *G. lamblia* are not associated with the season.

**Table 4.** Distribution of cases of protozoa according to year and season, and relation statistical tests.

	Frequency	%
**Rainy season (2020-1)**	(n=139) 83	59.71
2020	(n=67) 40	59.70
2021	(n=72) 43	59.72
**Dry season (2020-1)**	(n=48) 31	64.58
2020	(n=24) 19	79.17
2021	(n=24) 12	50.00
Chi-square	0.18049	
P Value	0.671	
*E. histolytica/dispar*		
Chi-square	0.12764	
P Value	0.7209	
*P. hominis*		
Fisher test		
P Value	0.5666	
*G. lamblia*		
Fishier test		
P Value	0.5081	

### Frequency curves of intestinal helminthiasis infections

**Figure 5.**
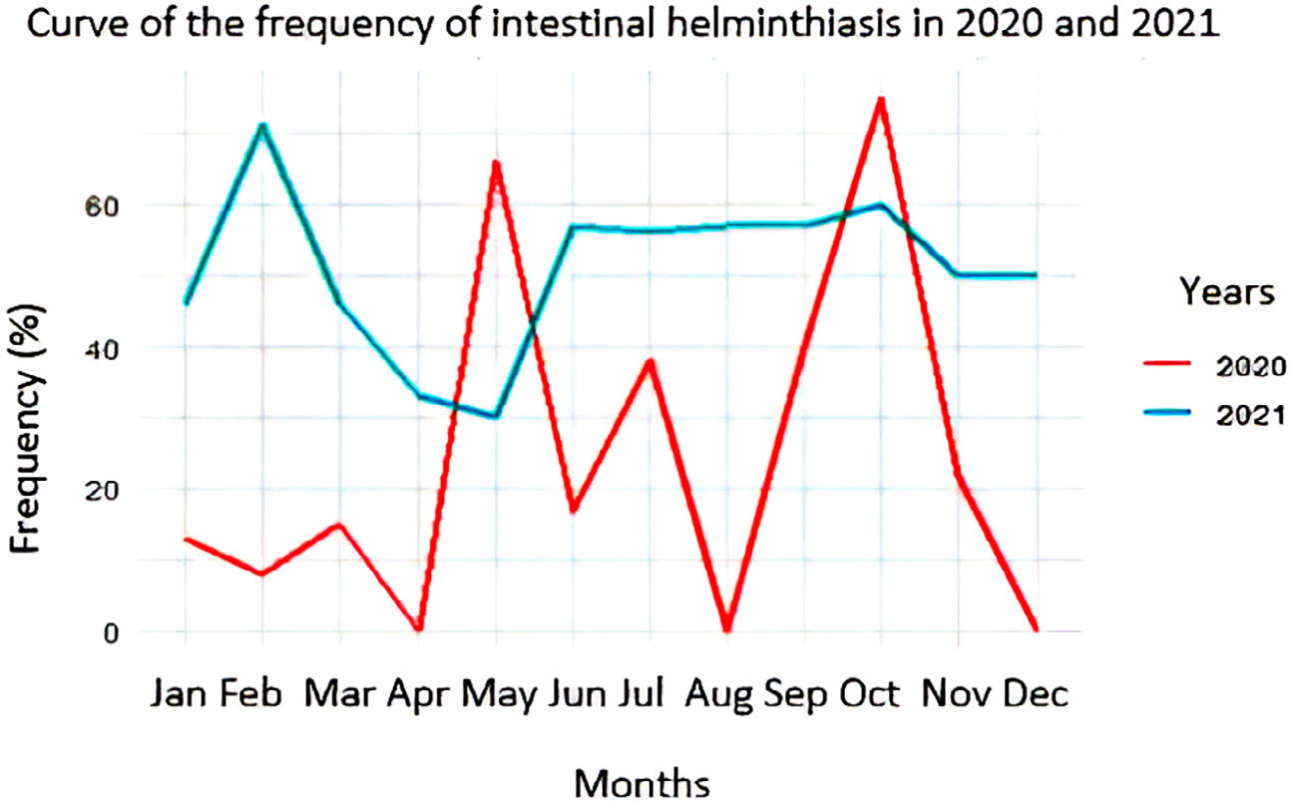
Curve of the frequency of intestinal helminthiasis in 2020 and 2021.

The frequency of intestinal helminthiasis was higher in 2021 than in 2020. In 2020, three high frequencies were recorded during the dry season (66% and 40% in May and July) and in October (74%). In 2021, three other high frequencies (72%, 56%, and 60%) were recorded in February, June, and October, respectively ( Figure 5).

### Intestinal helminthiasis prevalence and season relationship

The frequency of intestinal helminthiasis (37.50%) was higher during the dry season than during the rainy season (34.53%). In 2020, the dry season recorded a frequency of 25.00%, higher than that of the rainy season (17.91%). In 2021, both seasons recorded the same frequency (50.00%).

There is no association between the frequency of intestinal helminthiasis and the season. The frequencies of *A. lumbricoides*, *S. mansoni*, *A. duodenales*, and *E. vermicularis* are not associated with the season ( Table 5).

**Table 5.** Distribution of cases of helminthiasis according to year and season, and relation statistical test.

	Frequency	%
**Rainy season (2020-1)**	(n=139) 48	34.53
2020	(n=67) 12	17.91
2021	(n=72) 36	50.00
**Dry season (2020-1)**	(n=48) 18	37.50
2020	(n=24) 6	25.00
2021	(n=24) 12	50.00
Chi-square	0.038326	
P Value	0.8448	
*A. lumbricoides*		
Chi-square	9.4562	
P Value	1	
*S. Mansoni*		
Fisher test		
P Value	1	
*A. duodenales*		
Fisher test		
P Value	1	
*E. vermicularis*		
Fisher test		
P Value	0.4485	

## Discussion

In this study, we estimated the prevalence of intestinal parasitosis in general, as well as the prevalence of different types of intestinal parasitosis, among patients who underwent direct stool examination at CHUNDE in 2020 and 2021. These patients were aged between 2 years and 77 years, with an odds ratio of one woman for every man. In this study, the overall prevalence of intestinal parasitosis was high (75.40%). Intestinal protozoa (60.69%) were more frequent than helminths (35.29%). The most prevalent protozoa were *E. histolytica/dispar* (55.08%), *P. hominis* (9.09%), and *G. lamblia* (6.24%). The most common helminths were *A. lumbricoides* (27.81%), *S. mansoni* (3.74%), *A. duodenale* (1.60%), and *E. vermicularis* (1.07%). Polyparasitism was observed in 24.13% of patients, with the combination of *E. histolytica* and *A. lumbricoides* being the most frequent (14.44%). Intestinal parasitosis were particularly prevalent in children (95.00%) and elderly adults (88.89%), and more frequent among women (78.49%) than men (72.34%). Prevalence was higher, although no statistically significant association was observed between season and parasitosis prevalence.

The overall prevalence of intestinal parasitosis was 75.40%. The prevalence of intestinal protozoa and helminths was 60.69% and 35.29%, respectively. The most prevalent intestinal protozoa were *E. histolytica/dispar* (55.08%), followed by *P. hominis* (9.09%) and *G. lamblia* (6.24%). The most prevalent intestinal helminths were *Ascaris lumbricoides* (27.81%), followed by *S. mansoni* (3.74%), *A. duodenale* (1.60%), and *E. vermicularis* (1.07%). Worldwide, a study in Brazil estimated the prevalence at 10.8% (95% CI: 8.6–13.4). *Endolimax nana* was the most frequent parasite (4.8%), followed by *E. histolytica/dispar* (1.7%). In Africa, the prevalence of intestinal parasitosis remains high in many studies. Studies in Ethiopia and Morocco showed high frequencies of intestinal parasitosis but lower than ours, with prevalences of 52.9% and 43.18%, respectively. ^7, 14, 15^

In Guinea the prevalence of intestinal parasitosis is 52% with *A. Lumbricoides* (Figure 3), hookworm, and *S. mansoni* being the helminths most frequently found, with a prevalence of 15% (95% CI: 12,18), 6% (95% CI: 5,8), and 5% (95% CI: 3,7), respectively. Among protozoans, *E. histolytica* was predominant with a prevalence of 4% (95% CI: 3,5). The young children were less infected, with a prevalence of 37 % (95 % CI: 26,50) when compared to adults. The pooled prevalence of soil-transmitted helminth infections was 12%. ^16^

In the DRC, according to a 2019 study conducted in Kinshasa More than half of the diagnosed individuals were infested with *A. lumbricoides* at 58.3%, followed by 30% of individuals hosting *E. histolytica*, and *A. duodenales* was isolated in 11.7% of the diagnosed individuals. ^12^

In all these studies, the prevalence of intestinal parasitosis remains high, with intestinal protozoa being more frequent than helminths. We note some differences, that we attribute to the fact that these studies were conducted in different regions with varying hygienic conditions. Specifically, studies in Tunisia provide percentages of intestinal helminths and protozoa relative to the total number of people affected by intestinal parasitosis, while our study shows the actual prevalence of each specific parasite type within the total population studied.

45 patients (24.13%) were infested by multiple intestinal parasites simultaneously, with 19.79% combining at least one helminth and one protozoan. The most frequent combination was *E. histolytica* and *A. lumbricoides*, with 27 cases (14.44%). Other studies have noted cases of polyparasitism. A study conducted in Benin between 2005 and 2013 by Sissinto et al. showed a polyparasitism rate of 17.4% (3.09% of the total study population). The most common associations were *Blastocystis hominis* + *E. histolytica/dispar* (20.1%), *Entamoeba coli* + *E. histolytica/dispar* (17.2%), and *B. hominis* + *Endolimax nana* (11.7%). In Morocco, Zouitni reported a polyparasitism rate of 63.15% (27.2% of their study population). In the DRC, Woolf K. et al. reported a polyparasitism prevalence of 23.41%. We note similarities between our polyparasitism rate and those found in the last two studies mentioned. ^7, 10, 17^

Intestinal parasitosis were more frequent among children (95.00%) and elderly adults (88.89%). They were also more frequent among women (78.49%) than men (72.34%). In their study among children under 5 years old in Kivu, DRC, Woolf K. et al. recorded a prevalence of intestinal parasitosis of 94%, very close to the rate recorded in our study for children. In Guinea a meta-analysis showed a high prevalence of intestinal parasitosis among children under (37% of cases). A study in Bangui by Lango Y. et al. showed a higher prevalence of intestinal parasitosis in women (35.10%) compared to men (32.89%). ^3, 10, 16, 18^

There was no statistically significant association between season and the prevalence of intestinal parasitosis in general, nor with intestinal helminthiasis or protozoan infections. In the DRC, a study conducted in Sake (Kivu) among 504 children under 5 years old showed no association between season and the prevalence of intestinal parasitosis. ^10^ These results underscore the need to study the behaviors adopted by the population during different seasons to identify factors that directly affect the prevalence of parasitosis. As highlighted in the introduction, this region is known for its poor hygiene conditions, with frequent shortages of drinking water and poor waste management, especially for fecal matter.

The strength of this study lies in providing prevalence data for different types of intestinal parasitosis in Kasai Oriental, where such data had not been published until now. The weakness of this study lies in the fact that the direct stools examination differentiate *E. histolytica* from *E. dispar*; The techniques of enrichment of stools specific to certain parasites were not carried out and certain intestinal parasites, such as *S. stercoralis* and Schistosoma, require serological tests in addition to the examination of stools because they are often not detected by this examination. We believe that the prevalences of certain intestinal parasites could be underestimated. The small sample size and selection bias (hospital-based, symptomatic patients), limiting generalizability. The lack of data on confounding factors (socioeconomic status, water, sanitation).

The prevalence of intestinal parasitosis among symptomatic patients at CHUNDE, East Kasai, is considerably higher than reported in other African and international studies. This difference is due to the fact that the study took place in symptomatic patients. The predominance of protozoa over helminths is consistent with other studies, though the distribution of specific species varies by region. Polyparasitism was frequent and underscored the heavy burden of infection in this population. Seasonal variations were noted, but without statistical significance, suggesting that transmission is consistently high year-round.

## Conclusion

This study provides the first published prevalence data on intestinal parasitosis in East Kasai. The prevalence of intestinal parasitosis was higher among symptomatic patients at CHUNDE, with protozoa more frequent than helminths. Polyparasitism was common. There is no statistically valid association between the season and the prevalence of intestinal parasitosis.

## Author contributions

• **Conception and implementation:** All authors
• **Financial support:** All authors
• **Administrative support:** Lufuluabu M. Alphonse and Tshishimbi Jean Hubert
• **Provision of study material or patients:** All authors
• **Data collection and assembly:** Lufuluabu M. Alphonse and Tshodi B. Arsène
• **Data analysis and interpretation:** All authors
• **Manuscript writing:** All authors
• **Final manuscript approval:** All authors
• **Responsible for all aspects of the work:** All authors

## Disclosure statement

The authors are employees of the University of Mbujimayi, all in training at its medical faculty and assigned to its University Hospital Center Notre-Dame de l’Espérance.

## Ethics and consent

This study received approval from the ethics committee of the University of Mbujimayi on N52/CEUM1209 of 11th December 2019. It was conducted in accordance with the requirements of good clinical practices and the principles of the Helsinki Declaration of the World Medical Association, along with any subsequent relevant amendments. All patients who have participated in this study have provided their written informed consent by signing a written document, the consent form.

## Context

### Key findings

• The prevalence of intestinal parasitosis was high, at 75.40%.
• Intestinal amebiasis had the highest prevalence, at 55.08%.
• The prevalence of intestinal parasitosis was higher during the dry season.
• The prevalence was higher in children.

### Additional knowledge

• There is no statistically significant association between season and intestinal parasitosis.

### Global health impact on policies and actions

• Intestinal parasitosis are a major global public health problem.
• Intestinal amebiasis should be considered part of the Neglected Tropical Diseases (NTDs).
• In relation to SDG 6, there is a need to raise awareness and educate the population on hygiene.
• Funding longitudinal data collection is necessary to better understand the factors influencing the prevalence of intestinal parasitosis across seasons.

## Data Availability

Figshare: Impact of the season and prevalence of intestinal parasitosis at the Notre Dame de l’Espérance University Hospital Center. [Dataset]. *figshare.* 2023. https://doi.org/10.6084/m9.figshare.28050665.v1. ^19^ This study contains the following underlying data: Data.xls (anonymized results of microscopic examination of stools, yes=positive, no=negative, na=not attributable, p=rainy season, s=dry season, pro=protozoa, hel=helminth, adu=adult, enf=child, vieux=elder, *eh=E. hystolytica giar=G. lamblia, tric=P. hominis, al=A. lumbricoides, sm=S. mansoni, ank=A. duodenales, ox=E. vermicularis*). Data are available under the terms of the Creative Commons Attribution 4.0 International license (CC-BY 4.0).
