# Supplementary material for: Impact of the season and prevalence of intestinal parasitosis at the Notre Dame de l’Espérance University Hospital Center, Democratic Republic of the Congo
Source: F1000Res. 2025 Oct 6;14:50. Originally published 2025 Jan 9. [Version 3] doi: 10.12688/f1000research.160135.3 (PMC12511850; doi:10.12688/f1000research.160135.3)
Supplement: Peer review history

# Reviewer report: Reviewer response for version 3

**DOI:** 10.5256/f1000research.189165.r420835

**Published:** 2025-10-09

**Authors:** Ashok Kumar Sah

**Affiliations:** 1 A’ Sharqiyah University, Ibra, Oman

Thank you for your message. I have reviewed the revised manuscript and noted the authors’ responses and modifications. The revisions have appropriately addressed the concerns raised in my initial review.

 I am satisfied with the current version and recommend the manuscript for acceptance.

Is the work clearly and accurately presented and does it cite the current literature?

Yes

If applicable, is the statistical analysis and its interpretation appropriate?

Yes

Are all the source data underlying the results available to ensure full reproducibility?

Yes

Is the study design appropriate and is the work technically sound?

Yes

Are the conclusions drawn adequately supported by the results?

Yes

Are sufficient details of methods and analysis provided to allow replication by others?

Yes

Reviewer Expertise:

Medical laboratory science- Clinical chemistry, Pathology, diagnostics, carncer diagnostics

I confirm that I have read this submission and believe that I have an appropriate level of expertise to confirm that it is of an acceptable scientific standard.

# Reviewer report: Reviewer response for version 3

**DOI:** 10.5256/f1000research.189165.r420836

**Published:** 2025-10-09

**Authors:** Majed H. Wakid

**Affiliations:** 1 Special Infectious Agents Unit, King Fahd Medical Research Center, Jeddah, Makkah Province, Saudi Arabia

I noticed that there are no responses to my comments for Version 2, which included many major issues.

 The authors did not take any of my comments into  consideration  in Version 3.

 Please see my comments in Version 2 and amend the manuscript accordingly.

Is the work clearly and accurately presented and does it cite the current literature?

Partly

If applicable, is the statistical analysis and its interpretation appropriate?

Partly

Are all the source data underlying the results available to ensure full reproducibility?

Partly

Is the study design appropriate and is the work technically sound?

No

Are the conclusions drawn adequately supported by the results?

Partly

Are sufficient details of methods and analysis provided to allow replication by others?

No

Reviewer Expertise:

Professor and consultant with 30 years of experience in Diagnostic Medical Parasitology.

I confirm that I have read this submission and believe that I have an appropriate level of expertise to confirm that it is of an acceptable scientific standard, however I have significant reservations, as outlined above.

# Reviewer report: Reviewer response for version 3

**DOI:** 10.5256/f1000research.189165.r413874

**Published:** 2025-10-08

**Authors:** Sandra Noemi Escobar Arrieta

**Affiliations:** 1 Public university in Riobamba Canton, Riobamba Canton, Ecuador; 2 FACULTAD DE CIENCIAS, Escuela Superior Politecnica de Chimborazo (Ringgold ID: 27880), Riobamba, Chimborazo Province, Ecuador

An overview of the article

 Within an environment with limited resources, the manuscript "Impact of the season and prevalence of intestinal parasitosis at the Notre Dame de l’Espérance University Hospital Center, Democratic Republic of the Congo" tackles a significant public health concern. The study looks into how common intestinal parasite infections are in patients who have symptoms and whether there is any correlation between them and seasonal variation. All things considered, I think the subject is pertinent, and the manuscript offers useful information for the area. However, before the work can be deemed scientifically sound and appropriate for indexing, a number of methodological, analytical, and presentational concerns must be resolved.

 General remarks and suggestions

 1. The literature review, clarity, and presentation are just partially sufficient.

 I believe that further language correction would improve the paper. Many grammatical errors, inconsistent terminology, and inaccurate scientific names are present. For example, scientific names are not formatted in accordance with taxonomic norms, and terminology such as parasitoses and parasitosis are used interchangeably.

 In addition, several of the references that are mentioned are out of date—some of them are over 15 years old—and a number of them are either not in English or do not have all necessary citation information. I strongly advise adding recent studies (2020–2025) to the bibliography, such as more recent African studies and recent WHO reports. The article would also benefit from an improved introduction that highlights the knowledge gap in the Kasai Oriental region.

 2. The technique and study design are just partially sufficient.

 Although the study's sample method is advertised as random, it really involved successive sampling of individuals with symptoms who were referred for stool analysis. This limits how far the results may be applied and increases selection bias. This should be made explicit by the authors in the discussion and methods sections.

 Furthermore, the diagnostic method is restricted because it only uses direct wet mount microscopy. This method is insensitive and probably understates the actual prevalence. There were no molecular, concentration, or serological techniques employed, and it should be made clearer that E. histolytica and E. dispar cannot be distinguished from one another.

 Significant methodological information is also lacking, including the laboratory that conducted the analyses, the manner in which demographic data were gathered, and if any quality control protocols were adhered to.

 3. Reproducibility and availability of data: Only partially sufficient

 The work is missing a few important features that would enable complete reproducibility, even if the dataset is accessible on Figshare. These consist of details about sample handling, the staff members doing the analysis, quality control, and contextual elements like socioeconomic status or personal hygiene habits. It would be challenging for other researchers to replicate without these specifics.

 4. Interpretation and statistical analysis: Only partially sufficient

 Fisher's exact test and chi-square are suitable statistical techniques for fundamental analysis, but they don't account for potential confounding variables like age, sex, hygiene, or socioeconomic status. Additionally, the data and the interpretation of the results can occasionally be at odds.

 For instance, despite the fact that the results show no statistically significant correlation (p > 0.05), the discussion highlights that prevalence was higher during the dry season. The abstract, discussion, and conclusions should all be revised to correct this misunderstanding.

 5. Conclusions: The findings partially corroborate these conclusions.

 Other inferences, like the correlation with season, are not statistically justifiable, even though the data supports the conclusion that the prevalence of parasitic illnesses is high. Furthermore, important limitations such the limited sample size, diagnostic difficulties, and selection bias are not adequately acknowledged in the conclusions.

 It is also crucial to emphasize that the results cannot be directly extrapolated to the broader community because they are based on a sample of symptomatic patients from a hospital.

Is the work clearly and accurately presented and does it cite the current literature?

Partly

If applicable, is the statistical analysis and its interpretation appropriate?

Partly

Are all the source data underlying the results available to ensure full reproducibility?

Partly

Is the study design appropriate and is the work technically sound?

Partly

Are the conclusions drawn adequately supported by the results?

Partly

Are sufficient details of methods and analysis provided to allow replication by others?

Yes

Reviewer Expertise:

I have expertise in the following areas, which allow me to adequately assess the scientific content of this article: Parasitology, Basic and Clinical Microbiology, Food Microbiology, Cytology, Hematology, and Blood Analysis.

I confirm that I have read this submission and believe that I have an appropriate level of expertise to confirm that it is of an acceptable scientific standard, however I have significant reservations, as outlined above.

## References

[1] : Impact of the season and prevalence of intestinal parasitosis at the Notre Dame de l’Espérance University Hospital Center, Democratic Republic of the Congo. *F1000Research* .2025;14: 10.12688/f1000research.160135.3 10.12688/f1000research.160135.3 40657179

# Reviewer report: Reviewer response for version 2

**DOI:** 10.5256/f1000research.187631.r413877

**Published:** 2025-09-27

**Authors:** Ashok Kumar Sah

**Affiliations:** 1 A’ Sharqiyah University, Ibra, Oman

The manuscript is well-written, thoroughly analyzed, and presented in a clear and constructive manner. I would like to commend all the authors for their hard work and contributions. However, I have a few suggestions to enhance the scientific soundness and overall readability of the paper:

1. **Sample Size and Prevalence Rate**: The sample size appears to be quite small, yet the reported prevalence is notably high at 75.40%. It would strengthen the manuscript if the authors could discuss this apparent discrepancy—perhaps by providing justification for the sample size, addressing potential sampling bias, or contextualizing the prevalence rate within the broader population.
2. **References**: Several of the references cited are outdated. Updating the reference list to include more recent studies from the last five years would enhance the scientific rigor and relevance of the manuscript.

I believe addressing these points will improve the overall quality and impact of the study.

Is the work clearly and accurately presented and does it cite the current literature?

Yes

If applicable, is the statistical analysis and its interpretation appropriate?

Yes

Are all the source data underlying the results available to ensure full reproducibility?

Yes

Is the study design appropriate and is the work technically sound?

Yes

Are the conclusions drawn adequately supported by the results?

Yes

Are sufficient details of methods and analysis provided to allow replication by others?

Yes

Reviewer Expertise:

Medical laboratory science- Clinical chemistry, Pathology, diagnostics, carncer diagnostics

I confirm that I have read this submission and believe that I have an appropriate level of expertise to confirm that it is of an acceptable scientific standard, however I have significant reservations, as outlined above.

# Response

**Published:** 2025-10-01

**Authors:** lufuluabu mpemba Alphonse

**Affiliations:** Médecine interne, université de Mbuji-Mayi, Mbujimayi, kasai oriental, Democratic Republic of the Congo

Thank you very much for your comments.

 We have added them.

# Reviewer report: Reviewer response for version 2

**DOI:** 10.5256/f1000research.187631.r412656

**Published:** 2025-09-25

**Authors:** Majed H. Wakid

**Affiliations:** 1 Special Infectious Agents Unit, King Fahd Medical Research Center, Jeddah, Makkah Province, Saudi Arabia

***Please send a point-by-point response to each comment.***

**General Comments**

 - The authors didn't take most of my comments into consideration. I will add them again.

 - The suggested references (my articles) are very recent and relevant to the topic, and I have not received any regulations or objections from the journal regarding this.

 - The title in the “word” file does not match the title in the “pdf” file.

 - The authors still use “Mbuji-Mayi or “Mbuji-mayi” in their affiliations.

 - Additional references are still needed.

 - Some scientific names still need corrections, including the spelling or species presentation. [The genus name should be written in italics and capitalized, while the species name should also be written in italics but in lowercase. After the first mention, the genus name should be abbreviated to its first capital letter, also in italics, followed by a period and a space].

 - When et al is applied, only the family name of the author is used in addition to the year of publication. For example,

 - Most of the references are still written in a language other than English.

 - There is no “Conclusion” section at the end of the manuscript.

 - No limitations were added at the end of ‘Discussion’ section.

 - The author roles on page 2 are not the same as on page 11.

**Abstract**

 - Some scientific names spelling in the “Result” still need correction.

 - The conclusion is still not related to the study area.

**Introduction**

 - Line 3: Reference [2] is old, replace it with the suggested recent study [Al-Refai MF, Wakid MH: Prevalence of intestinal parasites and comparison of detection techniques for soil-transmitted helminths among newly arrived expatriate labors in Jeddah, Saudi Arabia. *PeerJ*. 2024; 12: e16820].

 - Reference 6 is not from the World Health Organization and is old (2008). A recent reference from a WHO publication or report should be added.

 - Reference 8 is not related to the 2016 Algeria study.

 - The presentation of some scientific names still needs correction including spelling.

**Methods**

 - The authors have not yet explained how they obtained the age and sex of the patients. Is there a questionnaire?

 - Which laboratory department received the samples and who performed the microscopic examination?

 - Macroscopic examination of the stool is very important to detect its color, consistency, and presence of adult worms or tapeworm segments. Have you performed it?

 - A direct stool examination alone is not a sufficient laboratory test for detecting intestinal parasites. Furthermore, the steps described above are completely unrelated to a direct smear. One gram of stool can’t be mixed with one milliliter of saline, a microscope slide, (The correct amount is 1 mg of stool and one drop of saline). Use the suggested reference: [Bahwaireth EO, Wakid MH: Molecular, microscopic, and immunochromatographic detection of enteroparasitic infections in hemodialysis patients and related risk factors. *Foodborne Path. Dis*. 2022; 19: 830-838.]

 - You can’t name the specific parasites you want to examine, as other parasites may be detected.

 - Written informed consent could not be obtained from the child. Please amend the sentence to read: "Written informed consent was obtained from the patients or their guardian, where applicable.".

**Results**

 - Correct “women/ woman” and “men/ man” to “female” and “male”, where applicable in the whole manuscript.

 - Lines 4-5: Unify the name of each “Age group” in the text, with Tables 1 and 2, and where applicable in the whole manuscript.

 - Remove the explanation of each age group in Table 1.

 - Table 1: Correct “Age groups” to “Age group”, and “childreen” to “Children”.

 - Delete “categories” from the x-axis in Figure 1, and Figure 2.

 - Table 2, some words were written in non-English letters.

 - Correct the scientific names ( *S. mansoni*) in Table 5.

 - What are (yes) and (no) used in Figures 1 and 2?  Present only positive cases.

**Discussion**

 - Correct the presentation of several scientific names.

 - Correct and modify the whole text in paragraph 2. The references (7,14,15) relate to Morocco, Brazil and Tunisia respectively. Add the reference number of the study discussed directly after it, not at the end of the paragraph.

 - Paragraph 3: correct to “In Tunisia, Cheikhrouhou et al. (2009)….”.

 - Paragraph 4: correct to “In the DRC, Nimo (2008)….”.

 - Correct and modify the whole text in paragraph 6. The references (7,10,17) relate to Morocco, DR Congo and Benin respectively. Add the reference number of the study discussed directly after it, not at the end of the paragraph.

 - Benin study from 2003 to 2015 not from 2005 to 2013.

 - Correct to “…by Sissinto-savi et al. (2018), showed….”.

 - Correct Woolf et al. to “Kapiteni et al. (2019),”.

 - Correct and modify the whole text in paragraph 7. The references (3,10,16,18) relate to Central African Republic, DR Congo, and Tunisia, respectively (3 and 18 for the same refrence). Add the reference number of the study discussed directly after it, not at the end of the paragraph.

 - Correct Woolf et al. to “Kapiteni et al. (2019),”.

 - Correct to “In Tunisia, Cheikhrouhou et al. (2009), showed….”.

 - Correct to “A study in Central African Republic by Lango-Yaya. et al. (2021), showed….”.

 - The limitations statement for this study has not been added yet. (Our findings are based on routine diagnostic procedures performed in CHUNDE on individuals referred to the study area. Therefore, despite its strategic geographic location and high patient population, these findings may not apply to the entire population. Furthermore, the stool samples in this study were not tested using any of the concentration techniques, which increases the possibility of detecting diagnostic stages of the intestinal parasites. Therefore, a major limitation of this study is the possibility that some intestinal parasite cases may be underreported due to the limitations of the direct wet examination [Reference: Alqarni AS, Wakid MH, Gattan HS. Prevalence, type of infections and comparative analysis of detection techniques of intestinal parasites in the province of Belgarn, Saudi Arabia. *PeerJ.* 2022; 10: e13889].).

**Add a “Conclusion” section after the “Discussion” section**.

**Data availability**

 Use suitable abbreviations for the corrected names of the parasites.

**References**

 - Most of the references are still written in a language other than English.

 - Reference No. 3 repeated in No. 18.

 - Data for most references is incomplete.

 - The references style does not comply with the journal's guidelines.

 - After adding suggested references/removing duplicate references, renumber in-text citations and references list.

Is the work clearly and accurately presented and does it cite the current literature?

Partly

If applicable, is the statistical analysis and its interpretation appropriate?

Partly

Are all the source data underlying the results available to ensure full reproducibility?

Partly

Is the study design appropriate and is the work technically sound?

No

Are the conclusions drawn adequately supported by the results?

Partly

Are sufficient details of methods and analysis provided to allow replication by others?

No

Reviewer Expertise:

Professor and consultant with 30 years of experience in Diagnostic Medical Parasitology.

I confirm that I have read this submission and believe that I have an appropriate level of expertise to confirm that it is of an acceptable scientific standard, however I have significant reservations, as outlined above.

## References

[1] : Molecular, Microscopic, and Immunochromatographic Detection of Enteroparasitic Infections in Hemodialysis Patients and Related Risk Factors. *Foodborne Pathogens and Disease* .2022;19(12) : 10.1089/fpd.2022.0024 830-838 10.1089/fpd.2022.0024 36067331

[2] : Prevalence, type of infections and comparative analysis of detection techniques of intestinal parasites in the province of Belgarn, Saudi Arabia. *PeerJ* .2022;10: 10.7717/peerj.13889 10.7717/peerj.13889 PMC937596535975233

[3] : Prevalence of intestinal parasites and comparison of detection techniques for soil-transmitted helminths among newly arrived expatriate labors in Jeddah, Saudi Arabia. *PeerJ* .2024;12: 10.7717/peerj.16820 10.7717/peerj.16820 PMC1082399038288465

# Reviewer report: Reviewer response for version 2

**DOI:** 10.5256/f1000research.187631.r413872

**Published:** 2025-09-23

**Authors:** Angela Bracho Mora

**Affiliations:** 1 Universidad Técnica de Manabí, Portoviejo, Ecuador

**In the abstract:**

 They must change the language “Intestinal parasitoses”

 I suggest not writing acronyms in this section. Writing full D.R

 If they only used conventional techniques, the way of writing is not *Entamoeba histolytica.*

*Pentstrichomonas hominis* is *Pentatrichomonas hominis*

*Ascaris lumbrucoide *is *Ascaris lumbricoides*

**In the introduction:**

 Change *Endolimax nanus*

 Change reference number 9 because it is very older.

 The correct form of *Schistosoma M *is *S. mansoni.*

 This sentence: *The prevalence of intestinal parasitic infections is likely very high at CHUNDE and may be associated with the season.* It’s isolated

 In the article it is not recommended to write specifics objectives

**In the results:**

 Entamoeba histolytica is not recommended to write because no used molecular techniques are used in the study.

 Leave a single title on the figures

**In the Discussion**

 There is a need for further discussion of the results since they are only compared with other works that are very old without giving their own interpretation and do not have a conclusion of the work

**General comments**

 The paper seems to be written in a language other than English, and it is necessary to check the translation as there are words that are not in this language.

 There are spelling errors that must be corrected and correct use of upper- and lower-case letters

 It has some old references that should be changed

Is the work clearly and accurately presented and does it cite the current literature?

Partly

If applicable, is the statistical analysis and its interpretation appropriate?

Partly

Are all the source data underlying the results available to ensure full reproducibility?

Yes

Is the study design appropriate and is the work technically sound?

Yes

Are the conclusions drawn adequately supported by the results?

No

Are sufficient details of methods and analysis provided to allow replication by others?

Yes

Reviewer Expertise:

Parasitology, Epidemiology

I confirm that I have read this submission and believe that I have an appropriate level of expertise to confirm that it is of an acceptable scientific standard, however I have significant reservations, as outlined above.

# Response

**Published:** 2025-09-24

**Authors:** lufuluabu mpemba Alphonse

**Affiliations:** Médecine interne, université de Mbuji-Mayi, Mbujimayi, kasai oriental, Democratic Republic of the Congo

Thank you for the feedback. 

 We Have incorporated it. For the titles of figures, it depends on the Journal.

# Reviewer report: Reviewer response for version 2

**DOI:** 10.5256/f1000research.187631.r412655

**Published:** 2025-09-18

**Authors:** Abayeneh Girma

**Affiliations:** 1 Mekdela Amba University, Tulu Awuliya, Ethiopia

General comments

 The study addresses a relevant public health issue in a resource-limited setting. It reports a very high prevalence of intestinal parasitosis (75.4%) and investigates an important potential environmental driver: seasonality. However, the manuscript in its current form has significant methodological flaws, presentation issues, and statistical limitations that substantially weaken its conclusions and impact. Major revisions are required before it can be considered for indexing.

 1. Abstract: The conclusion that "prevalence was higher during the dry season" directly contradicts the stated result of "no statistically valid association." This must be corrected to align with the statistical findings. Again, The term "Intestinal amebiasis" should be replaced with " *Entamoeba histolytica/dispar* complex" to reflect the diagnostic limitation accurately. Moreover, the specific prevalence rates should be formatted correctly (e.g., *E. histolytica*, *P. hominis*).

 2. Introduction: The background is generally good and establishes the public health importance of the topic. It could be slightly strengthened by more explicitly stating the knowledge gap for the specific region of Kasai Oriental.

 3. Methods, particularly on study design: Labeling it as "random sampling" is incorrect. Consecutive sampling of patients meeting the inclusion criteria is a better description.

 4. Discussion: The structure should be improved to flow from key findings -> comparison with literature -> interpretation -> limitations -> conclusion. Again, the discussion fails to adequately acknowledge the study's primary limitation: the diagnostic method. This should be the first point discussed. Additionally, comparisons with studies that used more sensitive diagnostic techniques are of limited validity and should be made with extreme caution. Moreover, the speculation about hygiene conditions is not supported by any data collected in this study.

 5. Limitations: The listed limitations in the manuscript are insufficient. The following must be added:

1.
  1. The use of direct smear only, leading to an underestimation of prevalence.
  2. The inability to differentiate *E. histolytica* from *E. dispar*.
  3. The small sample size and selection bias (hospital-based, symptomatic patients), limiting generalizability.
  4. The lack of data on confounding factors (socioeconomic status, water, sanitation).

6. Conclusion: The conclusions are not fully supported by the data. They should be tempered significantly: Instead of "The prevalence of intestinal parasitosis was higher," state: "A high prevalence of intestinal parasites was observed among symptomatic patients at this hospital, but the true community prevalence is likely different." Again, the conclusion on seasonality should clearly state that no statistically significant association was found, likely due to limited power and other unmeasured confounding factors.

 7. References: Some references are quite old (e.g., Faye 1998, and remaining two studies each conducted in 2008 and 2009). It should also be formatted uniformly according to the journal's guidelines.

Is the work clearly and accurately presented and does it cite the current literature?

Partly

If applicable, is the statistical analysis and its interpretation appropriate?

Partly

Are all the source data underlying the results available to ensure full reproducibility?

Yes

Is the study design appropriate and is the work technically sound?

Partly

Are the conclusions drawn adequately supported by the results?

Partly

Are sufficient details of methods and analysis provided to allow replication by others?

Partly

Reviewer Expertise:

Microbiology

I confirm that I have read this submission and believe that I have an appropriate level of expertise to confirm that it is of an acceptable scientific standard, however I have significant reservations, as outlined above.

# Response

**Published:** 2025-09-24

**Authors:** lufuluabu mpemba Alphonse

**Affiliations:** Médecine interne, université de Mbuji-Mayi, Mbujimayi, kasai oriental, Democratic Republic of the Congo

Thank you for the comments.

 We have integrated them. However, we did not change article number 4 because we only took an idea from it and not statistics on the prevalence of parasitoses that can vary.

# Reviewer report: Reviewer response for version 1

**DOI:** 10.5256/f1000research.175968.r399022

**Published:** 2025-08-27

**Authors:** Albert Abaka-Yawson

**Affiliations:** 1 University of Ghana, Legon, Ghana

ABSTRACT

 i. Authors should remove prevalence figures from background since they would be no opportunity to add reference

 ii. Methods section fails to talk about the study design and the sampling technique

 iii. You used "parasitoses" and "parasitosis" interchangeably in background and results section. Streamline that.

 iv. At the results section, parasites are presented as "Enterobius V., Schistosoma M. etc." consider rewriting them as "E. vermicularis, S. mansoni etc". In other words rather write the species in full and abbreviate the genus.

 v. Check spelling of various words throughout the manuscript. For instance, "Ancylostoma" not "Ankylostoma"

 MAIN WORK

 I. Remove general and specific objectives. Unless it aligns with journal's style

 ii. The methods section fails to capture the study design and sampling technique

 ii. Under results section, merge all 5 tables to one

 iii. Table 1: Remove ratio under sex

 iv. Authors should consider keeping just one. Either mean or median ages. Use only the best measure of central tendency

 v. Table 2: Under age, various categories are in French i.e., Enfants, Adultes etc. 

 vi. Discussion should not be bulletins and also reduce the numbers and repetition of results. Focus on discussing the results in the light of other studies as well as its implications on patients and future studies.

 vii. Also consider adding limitations to the latter part of the discussion

 GENERAL COMMENTS

 The manuscript is currently not in a indexing state but holds potential for improvement.

Is the work clearly and accurately presented and does it cite the current literature?

Partly

If applicable, is the statistical analysis and its interpretation appropriate?

I cannot comment. A qualified statistician is required.

Are all the source data underlying the results available to ensure full reproducibility?

Partly

Is the study design appropriate and is the work technically sound?

No

Are the conclusions drawn adequately supported by the results?

Partly

Are sufficient details of methods and analysis provided to allow replication by others?

No

Reviewer Expertise:

Neglected Tropical Diseases, Parasitology, Infectious Diseases Epidemiology, Laboratory Medicine

I confirm that I have read this submission and believe that I have an appropriate level of expertise to state that I do not consider it to be of an acceptable scientific standard, for reasons outlined above.

# Reviewer report: Reviewer response for version 1

**DOI:** 10.5256/f1000research.175968.r399028

**Published:** 2025-08-25

**Authors:** Majed H. Wakid

**Affiliations:** 1 Special Infectious Agents Unit, King Fahd Medical Research Center, Jeddah, Makkah Province, Saudi Arabia

**A- General Comments**

 - Mbuji-Mayi or Mbujimayi?

 - Additional references are required.

 - Scientific names need to be corrected throughout the manuscript (including Figures and Tables). The genus name should be written in italics and capitalized, while the species name should also be written in italics but in lowercase. After the first mention, the genus name should be abbreviated to its first capital letter, also in italics, followed by a period and a space. Thus, *Entamoeba histolytica* becomes *E. histolytica*. This must be done in both the abstract and the main text.

 - The name *Trichomonas intestinalis* is no longer used.

 - Some words were written in non-English letters.

 - There is no Conclusion section at the end of the manuscript.

 - There are some limitations to the study that should be mentioned.

 - In Author roles, delete “Funding Acquisition”, as there is no fund for this study.

 - Is it correct that the first letter of some authors name is written in lowercase?

 - D. R. Congo or DRC? also was abbreviated without the full name at first mention.

**B- Title**

 - Add the country name to the title.

**C- Abstract**

 - In the “Background” you mentioned that the prevalence in Africa is 42.18%, but in the Introduction section it is 43.12.

 - In the “objectives”, you must add the study area.

 - Correct all scientific names in the “Result”.

 - Use *Ancylostoma*, which is the preferred spelling, instead of *Ankylostoma*.

 - in the “Conclusion”, relate your conclusion to the study area.

**D- Introduction**

 - Line 2: correct to “As a result, they are rarely a reason for regular consultation and are often neglected”.

 - Replace reference [2], with a recent study [Al-Refai MF, Wakid MH: Prevalence of intestinal parasites and comparison of detection techniques for soil-transmitted helminths among newly arrived expatriate labors in Jeddah, Saudi Arabia. *PeerJ*. 2024; 12: e16820].

 - (WHO, 2023), should be given a reference number and added to the reference list.

 - Correct “ABOU BEKR BELKAID” to “Abou Bekr Belkaid”.

 - Correct all scientific names.

 - Include the objectives as a separate paragraph at the end of the Introduction section.

**E- Methods**

 - The authors didn’t mention the sample collection instructions provided to the patients.

 - The authors didn’t explain how they obtained the patients' age and sex. A questionnaire?

 - Which laboratory department received the samples and who performed the microscopic examination?

 - Macroscopic examination of the stool is very important to detect its color, consistency, and presence of adult worms or tapeworm segments. Have you performed it?

 - A direct stool examination alone is not a sufficient laboratory test for detecting intestinal parasites. Furthermore, the steps described above are completely unrelated to a direct smear. Two grams of stool equals approximately half a teaspoon, and when mixed with one milliliter of saline, it cannot be mixed on a microscope slide, (The correct amount is 1 mg of stool and one drop of saline). Direct iodine smear and permanent staining are also necessary methods to observe and confirm diagnostic features for identifying protozoan parasites. You can add this reference for these techniques (Macroscopic, Microscopic: direct saline, direct iodine, and permanent staining) [Bahwaireth EO, Wakid MH: Molecular, microscopic, and immunochromatographic detection of enteroparasitic infections in hemodialysis patients and related risk factors. *Foodborne Path. Dis*. 2022; 19: 830-838.]

 - There is no need to list the names of the parasites, as other parasites may be detected.

 - P value statistical significance was not mentioned in the statistical analysis.

 - Written informed consent could not be obtained from the child. Please amend the sentence to read: "Written informed consent was obtained from the patients or their guardian, where applicable.".

**F- Results**

 - Unify the name of each age group in the text, tables and figures.

 - Correct scientific names in the text, figures and tables.

 - Correct “women” and “men” to “male” and “female”, where applicable in the text, and tables.

 - The "Frequencies of intestinal protozoa" results section needs correction as in this section you represented results for helminths compared to *E. histolytica*.

 - The name “ *Trichomonas intestinalis*” is no longer used. Are you referring to *Retortamonas intestinalis* or *Pentatrichomonas hominis*?

 - Delete Figure 1, which adds no value to the results.

 - Table 2, some words were written in non-English letters.

 - Figures 2 and 3, are not in English, also these are not different types. What is yes and no used for?

 - Represent Figures 4, 5 and 6 in English.

**G- Discussion**

 - The discussion section should not contain subheadings.

 - Correct the presentation of the scientific names.

 - You should mention the limitations of this study, with the suggested reference: (Our findings are based on routine diagnostic procedures performed in CHUNDE on individuals referred to the study area. Therefore, despite its strategic geographic location and high patient population, these findings may not apply to the entire population. Furthermore, the stool samples in this study were not tested using any of the concentration techniques, which increases the possibility of detecting diagnostic stages of the intestinal parasites. Therefore, a major limitation of this study is the possibility that some intestinal parasite cases may be underreported due to the limitations of the direct wet examination [Alqarni AS, Wakid MH, Gattan HS. Prevalence, type of infections and comparative analysis of detection techniques of intestinal parasites in the province of Belgarn, Saudi Arabia. *PeerJ.* 2022; 10: e13889].).

**H- Add a Conclusion section.**

**I- References**

 - After adding the three suggested references, the total becomes 21 references.

 The references should be renumbered in the text citations and the references list.

Is the work clearly and accurately presented and does it cite the current literature?

Partly

If applicable, is the statistical analysis and its interpretation appropriate?

Partly

Are all the source data underlying the results available to ensure full reproducibility?

Partly

Is the study design appropriate and is the work technically sound?

No

Are the conclusions drawn adequately supported by the results?

Partly

Are sufficient details of methods and analysis provided to allow replication by others?

No

Reviewer Expertise:

Professor and Consultant in Diagnostic Medical Parasitology.

I confirm that I have read this submission and believe that I have an appropriate level of expertise to confirm that it is of an acceptable scientific standard, however I have significant reservations, as outlined above.

## References

[1] : Prevalence of intestinal parasites and comparison of detection techniques for soil-transmitted helminths among newly arrived expatriate labors in Jeddah, Saudi Arabia. *PeerJ* .2024;12: 10.7717/peerj.16820 10.7717/peerj.16820 PMC1082399038288465

[2] : Molecular, Microscopic, and Immunochromatographic Detection of Enteroparasitic Infections in Hemodialysis Patients and Related Risk Factors. *Foodborne Pathogens and Disease* .2022;19(12) : 10.1089/fpd.2022.0024 830-838 10.1089/fpd.2022.0024 36067331

[3] : Prevalence, type of infections and comparative analysis of detection techniques of intestinal parasites in the province of Belgarn, Saudi Arabia. *PeerJ* .2022;10: 10.7717/peerj.13889 10.7717/peerj.13889 PMC937596535975233

# Response

**Published:** 2025-08-26

**Authors:** lufuluabu mpemba Alphonse

**Affiliations:** Médecine interne, université de Mbuji-Mayi, Mbujimayi, kasai oriental, Democratic Republic of the Congo

Thank you very much for insightful comments.

 We will integrate them into the new version of our article.

 But we have a concern regarding the articles you are proposing to add. they are all your articles. wouldn't that be a matter of interest or is it allowed by the journal ?

# Reviewer report: Reviewer response for version 1

**DOI:** 10.5256/f1000research.175968.r399027

**Published:** 2025-08-23

**Authors:** Abayeneh Girma

**Affiliations:** 1 Mekdela Amba University, Tulu Awuliya, Ethiopia

General comments

 This study investigates the prevalence of intestinal parasitosis and its association with seasonal variation among patients at the Notre Dame de l’Espérance University Hospital Center in the Democratic Republic of the Congo (DRC). The authors report a high overall prevalence (75.40%), with *Entamoeba histolytica* and *Ascaris lumbricoides* being the most common parasites. The study also examines demographic and seasonal trends but finds no statistically significant association between parasitosis prevalence and seasons. While the topic is relevant to public health in tropical regions, the manuscript has several methodological and presentation issues that need addressing before it can be considered for publication.

**Major comments**

**I. Methodology and analysis**

1. **Sample size and representativeness:** The sample size (187 patients) is relatively small for a two-year study, and the inclusion criteria (only patients referred for stool examination) may introduce selection bias. The authors should clarify whether this sample is representative of the general population or specific subgroups (e.g., symptomatic patients).
2. **Seasonal analysis:** The definition of "dry" and "rainy" seasons is arbitrary (e.g., dry season: 15/5 to 15/8). The authors should justify this classification and consider regional climatic data.
3. **Statistical power:** The lack of association between season and parasitosis may reflect inadequate statistical power rather than a true absence of association. A power calculation should be included.
4. **Confounding factors:** The study does not account for potential confounders (e.g., hygiene practices, water sources, socioeconomic status) that could influence parasitosis prevalence independently of season.

**II. Results and Interpretation**

1. **Contradictory findings:** The abstract states that the prevalence was higher during the dry season, but the results section notes no significant association. This discrepancy should be resolved.
2. **Polyparasitism:** The high rate of polyparasitism (24.13%) is noteworthy, but the clinical or epidemiological implications are not discussed.
3. **Comparative data:** The discussion compares findings to studies in Tunisia, Morocco, and Brazil, but differences in study design, population, and diagnostic methods limit the validity of these comparisons.

**III. Presentation and clarity**

1. **Tables and figures:** Some tables (e.g., Table 2) are fragmented across pages, making them hard to follow. Figures (e.g., frequency curves) lack clarity in labeling and interpretation.
2. **Language and grammar:** The manuscript has numerous grammatical errors and awkward phrasing, which hinder readability. Professional editing is recommended.
3. **Data availability:** The dataset is available, but the description (e.g., "yes=positive, no=negative, na=not attributable") is unclear. A codebook or detailed metadata would improve reproducibility.

**Minor comments**

1. **Abstract:** The conclusion ("prevalence was higher during the dry season") contradicts the results ("no association"). Revise for consistency.
2. **Introduction:** The background could better highlight gaps in the DRC-specific literature.
3. **Discussion:** The high prevalence of *Entamoeba histolytica* should be contextualized with local diagnostic challenges (e.g., differentiation from *E. dispar*).

Is the work clearly and accurately presented and does it cite the current literature?

Partly

If applicable, is the statistical analysis and its interpretation appropriate?

Partly

Are all the source data underlying the results available to ensure full reproducibility?

Yes

Is the study design appropriate and is the work technically sound?

Partly

Are the conclusions drawn adequately supported by the results?

Partly

Are sufficient details of methods and analysis provided to allow replication by others?

Partly

Reviewer Expertise:

Microbiology

I confirm that I have read this submission and believe that I have an appropriate level of expertise to confirm that it is of an acceptable scientific standard, however I have significant reservations, as outlined above.

# Response

**Published:** 2025-08-25

**Authors:** lufuluabu mpemba Alphonse

**Affiliations:** Médecine interne, université de Mbuji-Mayi, Mbujimayi, kasai oriental, Democratic Republic of the Congo

Thank you very much for the comments on our article, **impact of the season and prevalence of intestinal parasitosis at the Notre Dame de l'Espérance University Hospital**.

 We will respond to your concerns in the order in which you listed them.

1.
  1. We did not conduct this study in the general population of the eastern region of the city of Mbujimayi served by the University Hospital, but rather at the University Hospital. The 187 subjects live in this region and consulted the University Hospital during the study period and they had symptoms that led the doctors to request direct examination of the stool. So it is a prevalence of intestinal parasitosis in symptomatic subjects who have consulted the University Hospital.
  2. The definition of the season is not arbitrary but rather very precise. Please see page 17 of this scientific document ( kasai-oriental.pdf). We will add this article to the revised version.
  3. And 4 Our study focused on the effect of climate on the prevalence of intestinal parasitosis. The finding is the lack of association, although on simple glance the data showed an increase in prevalence during the dry season. We plan to conduct a study in the general population including other factors to increase potency.

2.1 There is no contradiction. High prevalence during the dry season does not necessarily mean that there is an association between the dry season and prevalence. If this were the case, statistical analyses to prove an association would be useless. However, this lack of association shows the interest in looking for other factors that would normally influence this prevalence.

 2.2 This is an observation at this stage. Our study, which focused on the relationship between climate and the prevalence of intestinal parasitosis, does not have enough evidence to initiate such a discussion.

 2.3 Indeed, we even pointed this out in the discussion. These differences still exist but do not totally limit the comparison.

 3.1 It is a technical problem that will be fixed by the Journal. Our figures are clear and accepted by the Journal.

 3.2 We will reread our manuscript and correct any errors we find. However, please let us know for fear that these errors will still go unnoticed.

 3.3 There is indeed a code guide published with the data. Please check carefully.

 Minor comments

1. As explained in 2.1, there is no contradiction

Thanks for advices in 2 and 3.
